# From Focused Thought to Reveries: A Memory System for a Conscious Robot

**DOI:** 10.3389/frobt.2018.00029

**Published:** 2018-04-04

**Authors:** Christian Balkenius, Trond A. Tjøstheim, Birger Johansson, Peter Gärdenfors

**Affiliations:** ^1^Lund University Cognitive Science, Department of Philosophy, Lund University, Lund, Sweden; ^2^University of Technology Sydney, Ultimo, NSW, Australia

**Keywords:** working memory, semantic memory, computational model, episodic memory, consciousness

## Abstract

We introduce a memory model for robots that can account for many aspects of an inner world, ranging from object permanence, episodic memory, and planning to imagination and reveries. It is modeled after neurophysiological data and includes parts of the cerebral cortex together with models of arousal systems that are relevant for consciousness. The three central components are an identification network, a localization network, and a working memory network. Attention serves as the interface between the inner and the external world. It directs the flow of information from sensory organs to memory, as well as controlling top-down influences on perception. It also compares external sensations to internal top-down expectations. The model is tested in a number of computer simulations that illustrate how it can operate as a component in various cognitive tasks including perception, the A-not-B test, delayed matching to sample, episodic recall, and vicarious trial and error.

## 1 Introduction

### 1.1 The Inner World

Consciousness is not unitary but involves several kinds of components. The most fundamental component may be the emotional tone of the current state of the mind (Damasio and Marg, 1995). However, in this article, we will not consider emotions but focus on sensations that are the immediate sensory impressions, perceptions that are interpreted sensory impressions, and imaginations (or images) that are not directly governed by sensory impressions (Humphrey, 1992; Gärdenfors, 2003). After emotions, this is presumably the evolutionary order in which the different functions appear. Even for simple organisms, the sensory organs generate sensations. Perceptions require more advanced cognitive processing. The main function of perceptions is to provide information about the animal’s environment. Imaginations also require that sensations can be suppressed. The planning behavior of mammals and birds suggests that they have imaginations that concern entities not currently present in the environment.

On the first level, consciousness contains sensations. Our subjective world of experiences is full of them: tastes, smells, colors, itches, pains, sensations of cold, sounds, and so on. This is what philosophers of mind call qualia.

On the second level, an organism that in addition to bodily sensations is capable of representing what is happening at a distance in space or in time will be better prepared to act and thus improve its chances of survival. Several processes in the brain add new information to what is given by the sensations. This holds especially for the visual modality. For example, an object is perceived to have contours, but in the light that is received by the retina, there is nothing corresponding to such structures—this information is constructed by the visual process. By filling in extra information, perceptions help us choose more accurate actions.

On the third level, that of imaginations, sensory input is not used to trigger the filling-in processes, but they are initiated by inner mechanisms. An organism with imaginations can generate a prediction of the consequences of a particular action. Such simulations constitute the core of planning processes. The mechanisms involved in performing an action are the same as those in imagining a performance.

Imagining an action presupposes that the current sensations can be blocked, lest they conflict with the imagination. Glenberg (1997) writes that imaginations put reality in quarantine. The blocking is part of the executive functions mediated by the frontal lobes of the cortex. Glenberg (1997) distinguishes between “automatic” and “effortful” memory. The automatic memory is used to turn sensations into perceptions. For example, finding your way at home in the dark involves blending your limited sensations with your memories.

The effortful memory is used to create imaginations. What is called remembering is a special kind of image that is judged to correspond to an actual event. Effortful memory is also necessary for fantasies: a sphinx cannot be imagined unless you have previous memories of lions and humans.

Perceptions and imaginations taken together generate the “inner world” of an organism. Such an inner world is valuable from an evolutionary perspective. Craik (1967) writes: “If the organism carries a ‘small-scale model’ of external reality and of its own possible actions within its head, it is able to try out various alternatives, conclude which are the best of them, react to future situations before they arise, utilize the knowledge of past events in dealing with the present and future, and in every way to react on a much fuller, safer and more competent manner to the emergencies which face it.” For an organism with an inner world, actions are generated from a represented goal, rather than directly from the sensations (Jeannerod, 1994). This means that an organism that has imaginations has large advantages to one who must solve a problem by trial and error that can both be very inefficient and lead to dangerous situations. The inner world makes is possible for the organism to simulate different actions and evaluate their effects. Such simulations allow it to select the most appropriate action. Early evidence for such a process was presented by Tolman (1948), who showed that the searching behavior of rats in mazes is best explained by assuming that they have a “spatial map” as part of their imaginations.

An inner world is a *sine qua non* for consciousness. In this article, we will use two memory tests from research on infants as minimal criteria for deciding whether a system has an inner world: (1) exhibiting object permanence and (2) passing the “A-not-B” test (Piaget, 1954).

A child who exhibits object permanence understands that objects continue to exist even when they are not directly perceived. Piaget (1954) studied this by observing infants’ reaction to when a favorite object was hidden, say, under a pillow. According to him, object permanence develops between 4 and 8 months of age, but some researchers claim that it may develop earlier (Bower, 1974). Without object permanence an infant would not be able to identify an object or a person over time. It is considered to be a method for evaluating working memory in young infants.

In an A-not-B test, a toy is hidden under box A that is within the reach of an infant. The infant searches for the toy under box A and finds the toy. The hiding is then repeated several times. Then, in the test, the toy is hidden under box B that also is within the infant’s reach. Infants between 7 and 10 months typically make a perseveration error, looking under box A even though they saw the toy being hidden under box B. This behavior indicates that the infants have limited object permanence. When infants are 12 months or older, they normally do not make this error.

In this article, we present a novel memory system that supports the minimum operations for a conscious robot with the properties described earlier. The main function of this memory system is to move some cognitive operations into an inner world, and more importantly, to allow the inner world of the cognitive system to coevolve with the external world in such a way that it can generate expectations as those involved in object permanence and the A-no-B test. These expectations can be used in decision-making, to detect changes in the external world, and to direct attention. Furthermore, by allowing the inner world to become decoupled from external input, it can produce chains of “thoughts” based on semantic and episodic relations. Such chains can range from replay of previous episodes to novel combinations of previous experiences. In machines, an inner world in general and object permanence in particular promises to enable more robust goal directed action, visual search, and even planning.

We take a developmental robotics approach (Asada et al., 2009), and first want to model memory processes of the young infant, and later approach more complex abilities. Our goal here is to show how the proposed memory model supports many cognitive functions that are central to a conscious intelligent robot and to suggest that the model could form an important component of a larger cognitive architecture that will be tested in a robot in the future.

### 1.2 Models of Memory

One of the most canonical models of associative memory is the Hopfield network (Hopfield, 1982, 1984). The Hopfield network consists of a set of nodes connected by associations of varying strengths that store a set of patterns. The network operates as a content addressable memory where an incomplete activation pattern over the nodes will recall a complete stored pattern. An interesting aspect of the network is that it is possible to define an energy function that described every state of the network. It can be shown that the network changes its state in such a way that it decreases the energy of the whole system until it ends up in a local energy minimum. The minima of the energy function correspond to the stored memories. These states are attractors for the system in the sense that any initial state will move toward one these states. These types of networks lend themselves to model both perception and semantic memory but can also be extended to handle episodic associations by introducing delays on associations (Sompolinsky and Kanter, 1986). These properties are central to the model that we develop below and are used to process both semantic and episodic memories and to form associations that binds stimuli to places.

Cognitive operations also require working memory mechanisms and many computational models have been proposed. They emphasize different aspects of the working memory system, such as spatial map formation (Blum and Abbott, 1996), serial order recall (Page and Norris, 1998; Burgess and Hitch, 1999; Botvinick and Plaut, 2006), perseveration and distractibility (Kaplan et al., 2006), gating, action selection, and reinforcement learning (Ponzi, 2008), or sequence generation (Verduzco-Flores et al., 2012). One early computational model of working memory was proposed by O’Reilly et al. (1999). This model includes a prefrontal system that maintains contextual information that is used to bias different processes in the rest of the model. This is combined with a fast learning model of the hippocampus. Similar models were also described by Cohen et al. (1990) and Miller and Cohen (2001).

Focusing on the control aspect of working memory, Sylvester et al. (2013) describe a working memory system that controls the flow of information by opening and closing a network of gates. This system was used to do working memory cycling and comparison and was structured to adequately respond to *n*-back type tasks. Building on the gate paradigm, Sylvester and Reggia (2016) showed how a visual input could be associated with a location in the visual field to perform a card matching task. Both these systems rely on an instruction sequence memory (ISM) that can be programmed with sequences of gate configuration so as to respond adequately to the task at hand. The ISM consists of a Hopfield network (Hopfield, 1982) that can store attractor sequences by a mechanism of Hebbian learning (Hebb, 1949).

Moving away from cognitive and brain inspired models, more abstract neural network models have also begun to incorporate association mechanisms. For example, there has been a growing interest in adding external memory systems to deep-learning networks. In conventional deep-learning models, the memory of the network is stored implicitly in the entire network, in the form of unit weights. Hence, it is hard to store particular associations in such structures. This has prompted research into architectures that add external memory modules, allowing activation patterns to be stored alongside other data, such as labels, words, or sounds.

Most such memory modules, like the neural Turing machine (Graves et al., 2014) and the differentiable neural computer (Graves et al., 2016), evolvable neural Turing machine (Lüders et al., 2017; Parisotto and Salakhutdinov, 2017), have a form of key—value mechanism where the key is typically the output from another network structure like a convolutional or recurrent net. Depending on the sophistication, such memory modules can update based on evidence, learn ordering patterns, or supply answers to queries (Weston et al., 2014; Chen et al., 2015).

The memory system we propose here shares some properties with these models but is different in that it explicitly aims at roughly reproducing the properties of specific brain regions.

## 2 The Memory System

**Figure 1:**
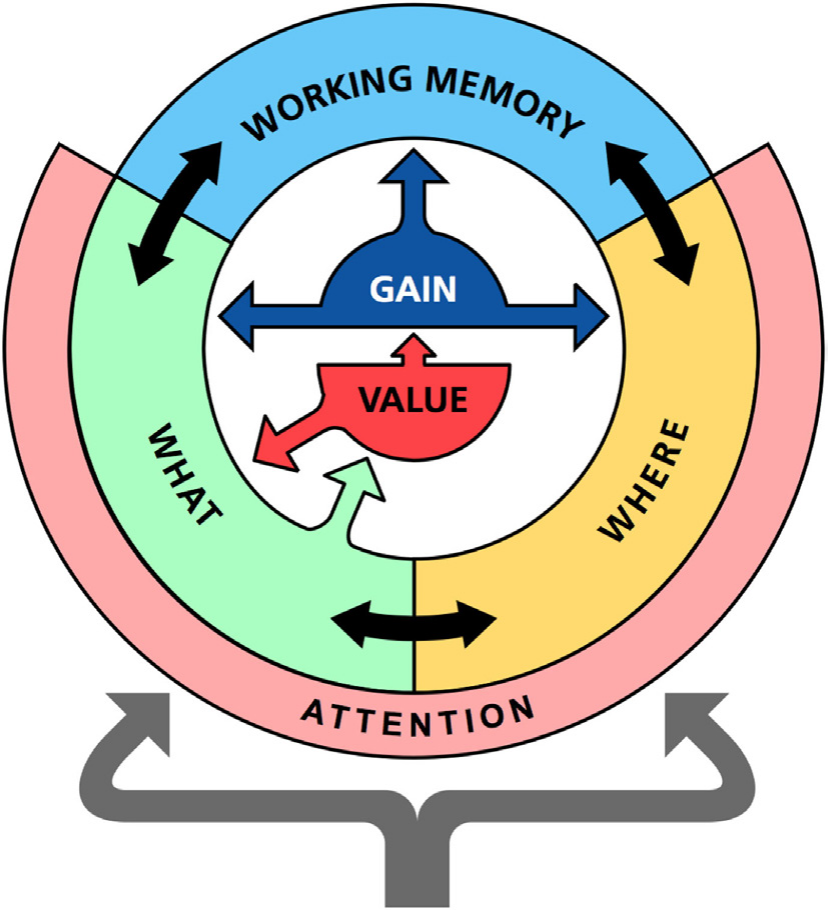
Overview of the memory model. The memory model consist of three main parts: the identification network (WHAT), the localization network (WHERE), and a prefrontal working memory network (WORKING MEMORY). Each network is modeled as a recurrent neuronal network with similar design but with slightly different dynamics. In addition to internal recurrent connections, there are also temporal associations that can read out sequences of states in memory. The identification and localization networks also include an attention component that detects novel external stimuli and compares expected to actual inputs to potentially generate surprise signals. The identification network communicates with value system (VALUE). All processing is under the influence of a gain modulation system (GAIN) that controls the randomness of the state transitions in memory.

This section describes the main components of the memory system and their functions. The model includes three interacting neural networks that roughly correspond to the ventral, dorsal, and prefrontal areas of the cortex (Figure 1). First, an identification network transforms sensations into perceptions; second, a localization network codes the spatial location of an object; and, third, a working memory network retains recently activated patterns over time.

### 2.1 Identification Network

The first component is the identification network that learns different stimuli as collection of stimulus properties. It corresponds to the WHAT system of the ventral cortex as proposed by Mishkin et al. (1983) and Goodale and Milner (1992). The part of this system that is included here can be sees as the highest level in a sensory processing hierarchy generating perceptions. It operates as a content addressable memory and recalls complete patterns based on partial inputs. We also assume that it generates top-down influence on sensory processing and interacts with value systems (Balkenius et al., 2009), but we do not model that here.

The identification, or WHAT, system is implemented as a fully connected network (see Appendix in Supplementary Material). This allows the network to settle into attractors that represent different memory states. In addition to the usual dynamics, we also include a mode of synaptic depression (Abbott et al., 1997; Tsodyks et al., 1998). This leads to a latching dynamics where the network can autonomously transition between different attractors (Lerner et al., 2010, 2012, 2014; Aguilar et al., 2017). This can be seen as free associations between the stored memory states (Russo et al., 2008; Akrami et al., 2012; Russo and Treves, 2012).

Furthermore, the identification network includes a comparator that compares the sensory input to the corresponding attractor state (Balkenius and Morén, 2000). Any stimulus or attractor component that differs contributes both to a total measure of surprise and to a feature-specific surprise that includes the parts of the sensory input that does not match the attractor state.

The current memory state is assumed to tune the attention system toward stimuli that match the state. For example, a state coding for the color red would tune the attention system to look for red objects in a way akin to the feature integration theory of attention (Treisman and Gelade, 1980). The identification network is thus assumed both to influence attention through top-down expectations, and to be influenced by bottom-up perceptual processes.

### 2.2 Localization Network

The second component is the localization network, or WHERE system. It parallels the functions of the parietal cortex (Andersen et al., 1985) and the hippocampus (Smith and Milner, 1981). Its role is to maintain a specific code for each possible location in the environment. This code is assumed to be activated when we look at a particular location.

It is similar to the identification network except that its activity is constrained by a winner-take-all-rule that implements the constraint that only one place is actively represented at each time. Associations between the identification and localization components allow the memory system to store bindings between places and objects. By associating each perceived object with its own individual location, the memory system avoids the binding problem where properties of different stimuli are mixed up in the network (ref). Another role of the localization network is that it increases the storage capacity of the identification component and avoids spurious attractors. The reason for this is that the localization codes are orthogonal for each location.

Like the identification network, this part of the memory model participates in both bottom-up and top-down processing. When we attend a particular location, the code for that location is activated in the localization network. Similarly, when a location code is activated by internal processes, it will influence attention and make us more likely to look at the coded location.

### 2.3 Working Memory Network

The final component is a “prefrontal” working memory (Fuster, 2009). The function of this network is to allow memories “stored” in working memory to be more easily recalled than other memories. According to our model, the actual working memories are not stored in the prefrontal system. Instead, the working memory function is the result of the interaction between prefrontal and sensory cortical areas. The working memory activation thus does not contain any sensory attributes although it is able to recall such attributes in the identification and localization networks (Lara and Wallis, 2015).

To allow the limited working memory to store any possible object–place binding, the nodes of this network are recruited when needed. The process is similar to that of an ART network (Grossberg, 1987), but less elaborate. The recruited nodes maintain an active state as long as the working memory is active. It is well known that prefrontal working memory cells operate in this way and allows for persistent activation during a memory period (Wang, 2001; Curtis and D’Esposito, 2003).

Each active working memory node can potentially influence the states of the identification and localization networks. Which node is allowed to do this depends on both the similarity of its learned input pattern and the current state of the complete system as well as the activity level of the node itself. The result of this mechanism is that a partial cue will recall the most recent state that is similar to the input.

The influence from the working memory network on the rest of the system involves both excitation and inhibition and can be likened to the inhibitory control exhibited by the prefrontal cortex (Fuster, 2009). Once a working memory node has been selected, it will promote the coding of its stored memory and inhibit other stimulus components (Desimone and Duncan, 1995). This can be seen as a top-down modulation of the states in the identification and localization networks (Gazzaley and Nobre, 2012). It can also indirectly control spatial attention through the localization network (Corbetta and Shulman, 2002).

### 2.4 Predictive Associations

In addition to the associations between the three networks, the memory system also contains predictive associations that work over time to predict the next state based on the current one. When allowed to run freely, these temporal associations will make the complete system transition between stable attractors over time in a way akin to daydreaming. When there is no input to the memory system, it will instead recall and internally play previously experienced sequences. As we will show below, this mechanism can be put to good use in choosing between different actions depending on their expected outcome. The predictive associations are learned in the same way as other associations except that there needs to be a delay between the activation of the two nodes that will be associated together. This will make the network to learn an association to the current state from a previous state of the network. The delay during learning is mirrored in a delay in the association that will be used to read out the prediction in the future.

### 2.5 Modes of Operation and Metaparameters

There are several parameters that can influence the operation of the memory system. The first is the level of noise. Memory transitions are highly dependent on the noise level and with sufficient noise; the state of the memory system will jump randomly between the different attractors. A moderate amount of noise allows the memory state to take new directions without being completely random, and a lower level makes the memory system more likely to stay in the same state for a longer time or to follow precise episodic memories.

In the brain, the locus coeruleus is believed to adjust the sensitivity to noise. This is a general arousal system and the main source of noradrenergic input to most of the brain. It has been suggested that the locus coeruleus, instead of changing the noise level, changes the response to noise by modulating the gain of cells involved in decision processes (Chance et al., 2002; Aston-Jones and Cohen, 2005; Donner and Nieuwenhuis, 2013; Eldar et al., 2013). Doya (2002) proposed that this should be seen as a metaparameter that allows the randomness of the processing to be controlled.

The second main parameter is the relative influence of the external input and internal expectations in controlling the memory state. The system can run in either in bottom-up mode where the internal state is controlled by external stimuli or in top-down mode where the sequence of memory states is internally produced. It is also possible to combine bottom-up and top-down processing. This allows the internal expectations to be compared with external stimuli and to make the system surprised when expectations are not met. Such a comparison also has an additional role. When there is a sufficiently large mismatch between the sensory input and the internal state, the memory system will be reset to allow the novel stimulus to quickly be coded in the different memory networks.

In the following sections, we apply the general memory system to a number of tasks and show how it can form the basis for many fundamental cognitive tasks. In these simulations below, the metaparameters were set heuristically to allow the model to show the desired properties in each case. When the memory system is used as a part in a complete architecture, these parameters are assumed to be learned for each particular task.

## 3 From Sensation to Perception

**Figure 2:**
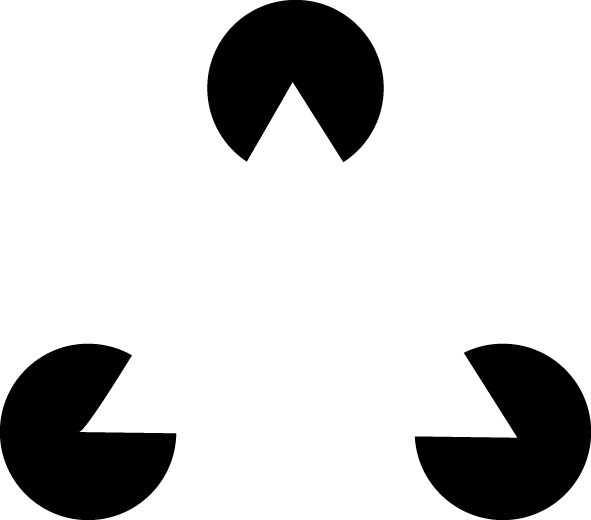
The Kanizsa triangle.

The role of perception can be seen when considering the well-known Kanizsa triangle (Kanizsa, 1976) (Figure 2). Our perceptions tell us that a white triangle lies on top of three black circles. Yet in the figure, there are no lines marking off the sides of the triangle from the white surroundings. The lines are a construction of our brains. There is a mechanism that simulates the existence of lines completing the segments of the circles.

Examples like this show that we have plenty of processes that complement the signals provided by the senses. Such complementations create the representations with which memory works—the perceptions, since what we remember is not only that which is presented by our sensory receptors but also that which is recreated, i.e., represented, by the filling-in processes. Here, we only consider a network with identical nodes and connections, but the reasoning is equally valid for more complex network. For example, Månsson (2006) developed a complex network that fills in contours in the Kanizsa triangle using a range of neuron models with different properties.

**Figure 3:**
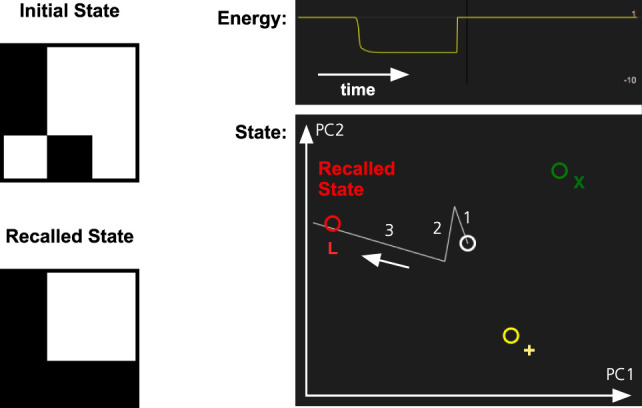
Pattern completion in the memory system. The memory has learned three patterns, L (red), X (green), and + (yellow). The partial activation of the L-pattern will make the memory system recall the complete pattern. The graph at the top right shows how the energy of the memory state decreases as the pattern in recalled. The graph below shows the memory state projected on a two-dimensional space defined by the first two principal components (PC1 and PC2) of the stored memory patterns. The graph shows the transition between an initial inactive state (white) and the recalled state (red). The numbers and arrow indicate the sequence of the different transitions.

In Figure 3, we illustrate how the pattern completion mechanism operates in the memory system. The system has learned three patterns, one of which is the letter L. When parts of the L are activated, the identification network will fill in the missing parts of it. In the figure, there are three stored patterns represented by different colors.

## 4 Object Permanence

A cat chasing a mouse that runs in behind a curtain can predict that it will come out the other side. So the cat can draw conclusions about the mouse even when it is receiving no direct signals from its senses. Such behavior presumes the cognitive ability called object permanence by Piaget (1954). This implies that the cat retains some kind of representation of the mouse even when its sensory impressions of the mouse are gone. The cat has expectations concerning the mouse.

Various studies of animals show that all mammals, birds, and octopuses possess object permanence. These organisms thus enjoy one more way to build in knowledge about the future in their consciousness. Object permanence is not innate, but it must be learned.

**Figure 4:**
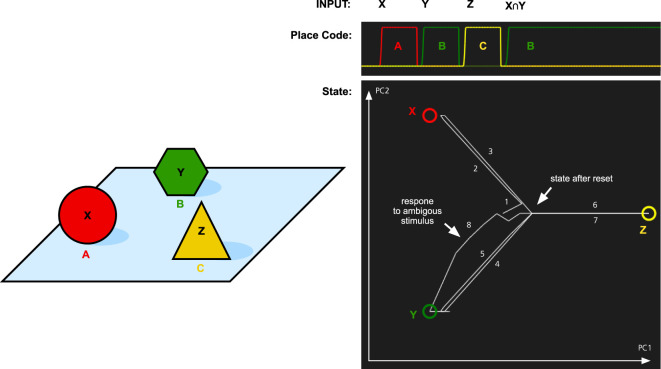
Simulation of place recall. Left: A scene with three objects X, Y, and Z at three places A, B, and C. The memory system was initially trained on these three objects. Right: The graph at the top shows the activation of the localization network when each object is used as input. Finally, an input pattern that consists of the overlapping parts of X and Y is used as input. This stimulus is equally similar to X and Y and thus ambiguous. The result is that the most recently attended place with an object similar to the input is recalled, that is, B. At the same time, the activity pattern in the identification system restores the complete pattern for Y. The graph at the bottom left shows transitions through the memory space. The image shows the memory state over time plotted in a two-dimensional space generated by the first two principal components (PC1 and PC2) of the attractor states. The circles represent the memories of X, Y, and Z, and the line shows how the memory state transitions between the memories as a response to the different input and the numbers show the order of the different transitions. The center of the image where all lines meet corresponds to the empty memory state after reset.

To test the memory model for its capacity to handle object permanence, we simulated two types of memory tasks. In both cases, the system is first presented with three objects X, Y, and Z. Each at its own location A, B, and C. In the first simulation, we tested if the memory system could recall the location of objects that it had previously seen (Figure 4). The memory was first cued with object X. This makes the memory state transition to the attractor for X. At the same time, the localization part of the memory system activates the location A that is associated with X. The locations are recalled for object Y and Z as well. Finally, we tested what is the result if we cue the memory with a stimulus that is similar to both X and Y. Here, we used an input pattern that contained only components that were shared by both objects. As can be seen in Figure 4, the memory state transitions to the attractor for object Y. The reason for this is that Y is more strongly coded in the working memory since it was seen more recently than X. In addition to showing the role of the working memory, this is also an example of pattern completion. The initial pattern is similar to both X and Y, and the memory state first moves toward a place between X and Y, before turning toward Y as more properties of Y are filled in.

**Figure 5:**
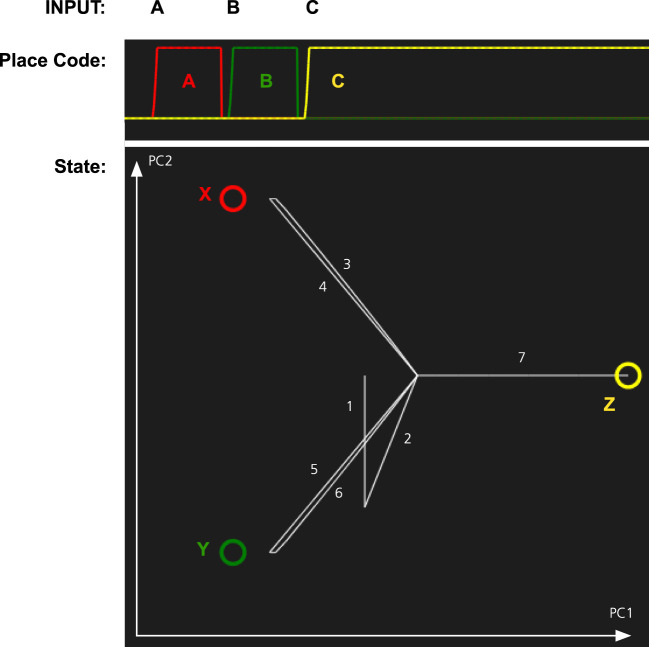
Simulation of the object recall for the scene in Figure 4. The memory system was first shown three combinations of objects and place: AX, BY, and CZ. Next it is cued with each of the locations A, B, and C. The graph at the top shows the activation of each place code over time. The graph at the bottom shows the path through the memory space as each location is cued. The state is initially wandering, which results in transitions 1 and 2 just before the system is cued with A.

In the second simulation, we tested whether the memory system can recall objects by being cued with locations. The results of this simulation are shown in Figure 5. When a location is cued, the memory state transitions to the attractor for the corresponding object illustrating that the memory system has formed expectations of which object is where.

The simulations show that the memory system can learn what object to expect at a particular location. Together with the comparator that compares expected and actual input, this allows the system to become surprised if expectations are not met (cf. Balkenius and Morén, 2000). It can also recall where it has seen an object. Such information can be used to determine where to search for an object and to direct the gaze while looking for it. The memory system thus has the essential properties needed for object permanence.

## 5 A-not-B

Another way to address object permanence is to run the A-not-B experiment on the memory model. To test if the memory system would make the A-nor-B error, we simulated the A-not-B task under two conditions. In the first, the output gain of the working memory system was low to simulate a brain at an earlier stage of development. In the second, the working memory gain was set at full strength. The system was first trained by repeatedly showing object X at location A. In the second step, we simulated moving object X to location B. This results in two stored memories in long-term memory, a stronger one that associates X with location A and a weaker one that associates X with location B.

**Figure 6:**
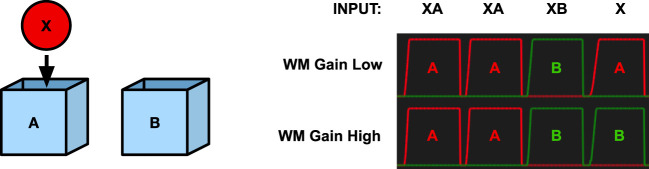
Simulation of the A-not-B task. Left—The object X and the two boxes A and B. Right—The graphs show the activation of the place code with low or high working memory gain for place A (red) and B (green), respectively, as response to different inputs. X represents the object stimulus, and A and B represent the two boxes. The final input X corresponds to the questions “Where is X?” With an undeveloped prefrontal cortex (low working memory gain) the model replies A. With a developed prefrontal cortex (high working memory gain), the model replies B.

To test the system, we activate the pattern for X in the WHAT system and allow the system to activate a location code in memory. When the working memory is turned off, the stronger association will win, and the system will recall location A (Figure 6). However, when the working memory system is turned on, the result is different. In this case, the working memory will remember each perceived stimulus. Every time a new stimulus is perceived, a new node in working memory will be activated while the activity in the remaining working memory nodes will decay slightly. As a consequence, a number of stimuli can be held in working memory at the same time. When a pattern is activated in the WHAT or WHERE components, the working memory cooperates to fill in missing information. Here, the perception of the stimulus X will recall the most recent activation containing X and read out its location B, thus avoiding the A-not-B error (Figure 6).

The performance of the models can be related to the serial position effect (Murdock, 1962). The initial error can be seen as a primacy effect, where the initial location of the object is stronger in memory as a result of multiple presentations. The avoidance of the error can be seen as the results of a recency effect, where the most recent location is more easily recalled. This view is in line with the model by Munakata (1998) that suggests that the A-not-B error is a result of competition between latent and active memory traces. However, the behavior of the model is different from the usual recency effect since it depends on a working memory component and not on short-term memory.

Our results fit well with findings suggesting that working memory is a driving force in cognitive development (Kail, 2007). An alternative theory of the A-not-B error is that it depends on the strength of the initially reinforced response to search at A (Diamond, 1998). We do not exclude that such a factor could also be involved, but our simulation shows that a working memory explanation may be sufficient. However, the working memory here influences the rest of the system by inhibiting the incorrect location, and similar mechanisms could presumably be used to inhibit an incorrect response in a similar way to an incorrect location.

## 6 Delayed Matching to Sample

The delayed matching to sample task (DMTS) is a variant of more general delayed response tasks (Rodriguez and Paule, 2009). Such tasks involve the presentation of stimuli, followed by a delay where no stimuli are given. The original stimulus is then presented along with one or several choice options, and the subject is required to choose which matches the original.

The task can be varied in difficulty by changing the delay time, or by altering the number of options to choose among during the response. Distractors may also be introduced to affect subjects’ ability to maintain attention and to impair working memory capacity (Rodriguez and Paule, 2009). Lesion studies in monkeys (Gaffan and Weiskrantz, 1980) indicate that the prefrontal and inferior temporal cortices are involved in DMTS tasks. Specifically, performance for tasks with visual stimuli is impaired after a higher visual area of the inferior temporal cortex has been damaged. Lesioning the prefrontal cortex appears to reduce the delay after which a correct response can be made but does not impair successful completion as such (Mishkin and Manning, 1978).

The configuration of the visual stimuli may take different forms, depending on which specific aspect of memory is under scrutiny. Sawaguchi and Yamane (1999) used a white square presented at one of four peripheral positions, placed equidistantly about a central focus point to study spatial memory. Tanji and Hoshi (2001) used a more complex setup with three cues placed in a pyramid pattern, each showing either a circular or triangular shape. This was used to study behavioral planning based on shape or location matching. Other variations of the DMTS task have been used to study color matching (Mikami and Kubota, 1980; Giurfa et al., 2001), movement matching (Ferrera et al., 1994), and horizontal vs. vertical orientation matching (Giurfa et al., 2001). The simplicity of the task makes it suitable for studying memory effects across various species, including humans (see, e.g., Daniel et al. (2016) for a review).

**Figure 7:**
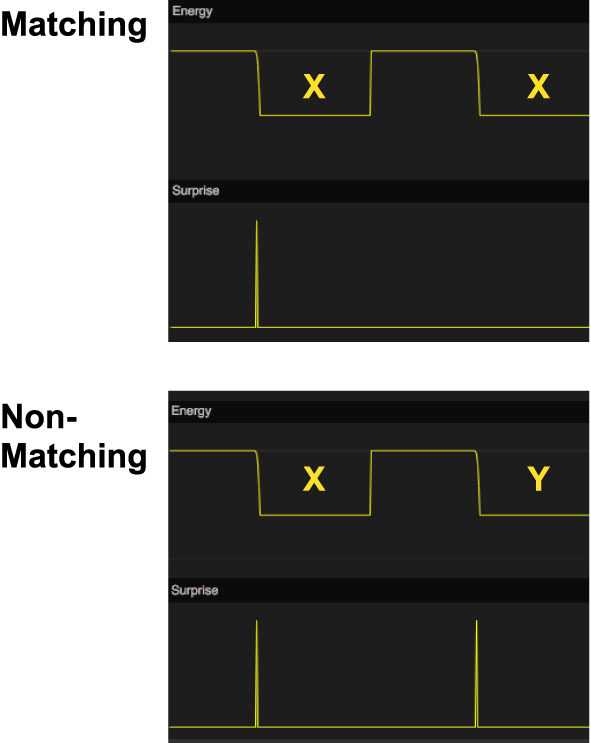
Simulation of delayed matching to sample (DMTS). In the top graph, the stimulus X is first shown as a sample stimulus and is subsequently followed by X again as comparison stimulus. There is no surprise signal the second time X is shown, indicating that the model recalls that it has seen this stimulus before. In the bottom graph, the sample stimulus X is followed by comparison stimulus Y instead. In this case, there is a surprise signal for the non-matching stimulus. The energy function is used to show the timing of the stimuli.

Using our memory model, we simulated a delayed matching-to-sample task (Figure 7). The system is first presented with a sample stimulus X that it will store in working memory. After a delay period, a comparison stimulus, X or Y, is presented. For each stimulus, the working memory network will read out the remembered stimulus and compute the match to each of the comparison stimuli. We assume that there exists a mechanism external to the memory system that selects the stimulus that generates the least surprise.

Our simulation shows that the memory system has the necessary memory functions for a delayed matching-to-sample response.

## 7 Daydreaming and Episodic Recall

Two possible mechanisms are involved in producing transitions between attractors. The first is the noise in the system that can kick the network out of an attractor if it is strong enough. The second mechanism is synaptic depression that weakens synapses that are involved in maintaining the current attractor. This has the effect of eventually making the state wander away from the attractor. A possible interpretation is that this is what occurs when the attentional system is not engaged, which makes the memory system enter a state of daydreaming where it can wander freely. The mind wandering produced by the model does not have any function but is instead a natural consequence of the function of the memory system. This is in line with the view presented by Mason et al. (2007) who suggest that the mind wanders “simply because it can.”

Herrmann et al. (1993) distinguish between semantic and episodic transitions in neural networks. Semantic transitions occur between states that are semantically related and are caused by synaptic depression that moves the state away from one attractor in favor of another one with overlapping activation pattern. Episodic transitions, on the other hand, are caused by predictive temporal associations (Sompolinsky and Kanter, 1986).

**Figure 8:**
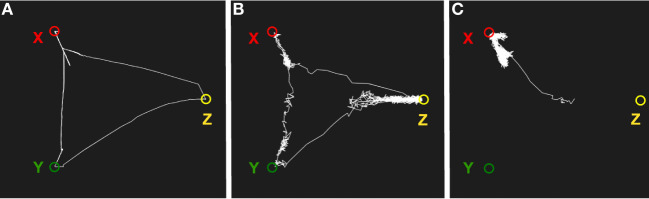
Simulation of mind wandering using semantic associations. **(A)** With low noise, the system will transition between semantically related states as a result of synaptic depression. **(B)** With a higher noise level, the memory system will transition less regularly and can potentially end up in semantically unrelated states. **(C)** With low synaptic depression, the system will move away from an attractor but return back again most of the time.

Figure 8 shows a simulation of semantic associations in the memory system. The system was first trained with three patterns X, Y, and Z where X and Y share some features, Y and Z share some other features, but X and Z do not share any features. With low noise, the system transitions randomly between X and Y, and between Y and Z, but not between X and Z. With a higher level of noise, the transitions occur between all states. Finally, with no noise, the system returns to the same state after synaptic depression. Although we want to like this wandering to daydreaming, it is obviously limited to combinations of states that the network has previously experienced.

**Figure 9:**
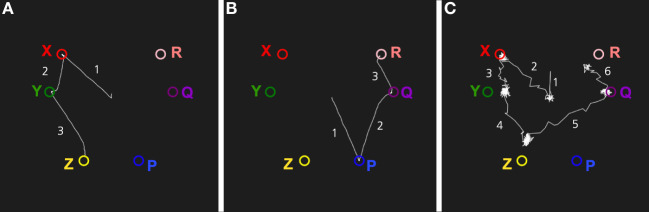
Simulation of episodic recall. The three graphs show transitions between the attractors of the network. **(A)** Recall of the episode X, Y, Z cued by an input X. **(B)** Recall of the episode P, Q, R cued by P. **(C)** A higher noise level produces a novel imagined episode that is a combination of two experienced episodes: X, Y, Z, Q, R.

Figure 9 shows a simulation of episodic recall in the memory system. The system was first trained with two sequences of stimuli: X, Y, Z and P, Q, R. When presented with X as an input, the memory system will read out the sequence X, Y, Z (Figure 9A). Similarly, for an input P, the sequence P, Q, R will be produced. When the noise level is increased, the episodic recall will sometimes transition from Z to Q, producing a novel sequence X, Y, Z, Q, R (Figure 9B). This shows how the memory system can combine two episodes into a novel imagined episode. The evolutionary value of such reveries is that they allow the memory system to generate new combinations of memories that can form the kernels for new plans. Some of these plans can be tried out at later occasions. Hence, the same mechanism that produces daydreaming can be seen as an element in a generate-and-test procedure.

## 8 Vicarious Trial and Error

**Figure 10:**
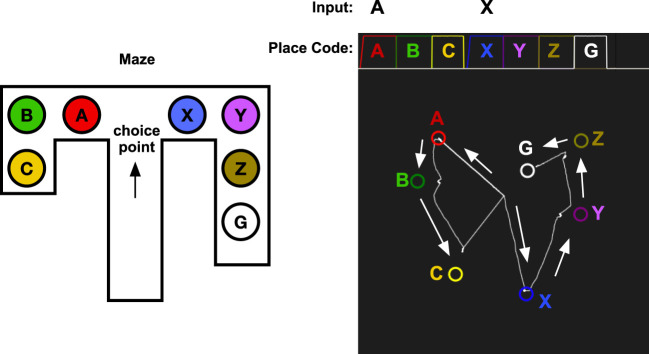
Vicarious trial and error. The memory system is assumed to have learned the sequence of places that are experienced while traveling through the maze. At the choice point, the memory system is used replay the result of choosing A or X. When looking left toward A, the memory cued with A and will start to replay A, B, C. When looking right, the memory system is cued with X which will replay the sequence X, Y, Z, G, which leads to the goal. The graph in the top right shows the activation of the place codes on the localization network. Note that the activation of A and X is slower as they are cued by an external stimulus. The graph in the bottom right shows the transitions through the identification network. The state starts at the center as A or X is received and moves through the states for the different places in the maze. The arrows show the direction of the memory transitions.

If an agent has an internal model of the world, it can make simulations of the consequences of actions (Craik, 1967). Redish (2016) proposes that animals internally simulates the outcomes of different choices before making the choice in the external world. As noticed by Muenzinger (1938) and Tolman (1939), rats look back and forth at different alternatives at a choice point. A rat that has to choose whether to go left or right in a maze can use its episodic memory to simulate selecting the left or the right path (Figure 10). The episodic memory recall described earlier is ideally suited for this process. By cueing the memory system with the stimulus A to the right, the sequence of moving through A, B, and C will be simulated internally. When looking right to see X, the sequence X, Y, Z, G will be produced instead. Since this sequence leads to the goal, the rat can now chose to go right.

Figure 10 shows a simulation of vicarious trail-and-error in a simple maze. The memory system has first experienced moving through the maze along two different routes. The first consists of locations A, B, and C which is a dead end, and the other consists of the sequence X, Y, Z, which finally leads to the goal G. At a choice point in a maze, the robot can look left or right, and the memory system is used to imagine the result of select one of the two possible paths. Looking at A, which will read out the sequence A, B, C that does not lead to a goal, and looking at the second alternative X, will read out the sequence X, Y, Z, G, which ends with the goal. This mechanism could be used by a decision mechanism that chooses between alternative actions based on their expected consequences.

Redish (2016) suggests that this type of mechanism is responsible, not only for spatial navigation but also for deliberative processes in general and that the internal schema used to simulate the world is what (Tolman, 1948) would call a cognitive map. This view of the cognitive map is in line with Tolman’s original view where the cognitive map did not have to be spatial but could be used for any kind of problem solving. Our proposed memory model can thus operate as a cognitive map that supports elementary planning operations.

## 9 Discussion

We have introduced a memory model for robots that can account for many aspects of the presence of an inner world, ranging from object permanence, episodic memory, and planning to imagination and reveries. It is modeled after neurophysiological data and includes many parts of the cerebral cortex together with a model of the arousal system. It consists of three main components, an identification network, a localization network, and a working memory network. An important aspect of the model is that the mechanisms that fill in sensations to generate perceptions can be detached from sensory input and run in isolation (Gärdenfors, 2003). This allows for planning mechanisms and for daydreaming that can serve as an investigation of a space of possibilities as a preparation for generating plans.

We propose that a robot equipped with this memory system together with mechanisms for more advanced sensory processing and action selection would have the required cognitive equipment to produce a basic form of consciousness—at least to the extent that it can be tested in behavioral experiments. A fundamental aspect of this model is that consciousness in not something that has to be added to the cognitive system. Instead, it is something that occurs naturally once a memory system is able to fill in sensory information and produce memory transitions over time. This will create an inner world that is used both to interpret external input and to support thoughts disconnected from the present situation.

The memory system can operate either in bottom-up mode, where external input directly controls the internal state, or in top-down mode, where previously experienced episodes control the progression of internal states. The internal flow of thoughts is modeled as transitions between memory states. The randomness of these transitions depends on the input from the locus coeruleus. In one extreme, the memory state is stuck in the current attractor, but when the sensitivity to noise increases, the memory state will start to transition to semantically similar states—also supported by synaptic depression. At the same time, episodic associations between states will make the memory replay sequences of states that it has previously experienced. When the randomness increases further, the memory state can make transitions between increasingly unrelated states. The locus coeruleus input thus acts as reins for focusing thought and thus preventing the system from ending up in galloping reveries.

It is an open question how the randomness of the memory processes should be controlled to optimally utilize the memory system for different tasks. Here, we did not include other parts of a complete system that could operate on the memory system. One interesting addition would be to add a reinforcement learning system that could learn to control the level of noise in the memory system to control transitions between different attractors (Lerner and Shriki, 2014). Such a reinforcement learning system could potentially control the various metaparameters to adapt the memory processing to the task at hand (Doya, 2002).

Another addition would be to allow a reinforcement learning system to control the different memory operations, in particular the storage and read out from working memory. In the current model, working memory is not controlled explicitly but stores every memory state as it occurs. From a developmental perspective, this is a reasonable approach before efficient utilization of working memory has been learned and constitutes a substrate for future learning of internal memory operations.

The memory system presented in this work can be contrasted with that described by Sylvester and Reggia (2016). The main difference between their work and ours is first the employment of gates, and second the inclusion of a discrete control module to sequentially set configurations of those gates. There is also a difference in the way the systems learn. Sylvester and Reggia (2016) explicitly program their system by imposing attractor states on a sequence memory part of the control module. By contrast, our system learns sequences of states from observation. Hence, Sylvester and Reggia (2016) can be likened to a system being taught by a teacher, while our system learns by discovery. Both systems utilize Hopfield networks for storing attractor states and employ forms of working memory. In our case, although the working memory does not store visual patterns as such, only associations between high-level sensory representations. The nature of those representations is arbitrary, but we chose to focus on object identity and location for this work. We do, however, acknowledge the utility of gating mechanisms for learning action sequences and plan to incorporate such mechanisms in future models.

Another important next step will be to test the model on a humanoid robot. We will use visual input from cameras that will be analyzed through a bidirectional deep-learning network before reaching the identification network described here. Similarly, the localization network will receive input that uses a population code for locations in three dimensions in several coordinate systems. A robotic implementation already exists with a minimal version of each of these components, but further development of the sensory processing is needed before the experiments simulated here can be tested in a robot in a natural environment.

When the internal processes meet the external input, the memory system is used to compare expectations against the external world to potentially produce surprise and control action selection. We did not include mechanisms for action selection here, but the output from the comparator of the attention system could easily be used for such selections. For example, to learn delayed matching or non-matching to sample, an action selection system would only have to associate the output of the comparator with selecting to refraining from selecting a particular stimulus. Similarly, to choose the correct path through a maze, the mechanism for vicarious trial and error we demonstrated would need to be interfaced with an action selection mechanism that learns to evaluate alternatives and select the one that leads to the goal. Given that the memory system does most of the work, very little remains to be learned by an action selection system.

Attention plays a crucial role as the interface between the inner and the external world. It directs the flow of information from sensory organs to memory and in the other direction it is responsible for the top-down influences on perception. The internal and external world can be seen as two dynamical systems that can be coupled or decoupled in different ways depending on the state of the organism and the task at hand. This allows the proposed model to bridge the gap between cognition as internal processing and situated cognition. We suggest that during evolution, as well as during the development of an organism, one finds a gradual change from acting in the external environment to operating in an internal world.

When the flow of thought through the inner world is cued by the immediate external stimuli, the memory system is used to evaluate the consequences of different available options. When allowed to flow freely, there need not be any relation between the train of thought and the current situation, but by changing the balance between bottom-up and top-down processing, the system can quickly be dragged back to the present situation. On the other hand, when the bottom-up influence is low, the system will start to daydream and replay experienced episodes or producing novel never experienced episodes by combining memories in new ways. The new combinations can then be used as input to the planning mechanisms. The same mechanisms are thus used both for focused goal-directed thought and for daydreaming and reveries.

## Author Contributions

All authors contributed equally to this work.

## Conflict of Interest Statement

The authors declare that the research was conducted in the absence of any commercial or financial relationships that could be construed as a potential conflict of interest.
