# Supplementary material for: From Focused Thought to Reveries: A Memory System for a Conscious Robot
Source: Front Robot AI. 2018 Apr 4;5:29. doi: 10.3389/frobt.2018.00029 (PMC7805698; doi:10.3389/frobt.2018.00029)
Supplement: Supplementary file 1 [file presentation_1.PDF]

Supplementary Material: From focused thought to reveries: A memory system for a conscious robot

Christian Balkenius, Trond A. Tjøstheim, Birger Johansson and Peter Gärdenfors

*Correspondence:
Christian Balkenius, Lund University Cognitive Science, Box 192, 221 00 Lund, Sweden
christian.balkenius@lucs.lu.se

APPENDIX

This appendix gives an overview of the current implementation of the model. The main parts are illustrated in Fig. 1. The WHAT, WHERE and WORKING MEMORY components all consist of fully connected networks, but the internal dynamics is different for each of the components. Every node in each of the networks receives input from all the nodes in the other networks (indicated by the black arrows in Fig. 1). Each component is described below in this appendix. For further details, the reader is referred to the source code that is available at www.ikaros-project.org.

What

The what-component consist of a fully connected recurrent network. The activity x_j of each node in the network at time t is changes according to

$$\dot{x}_j(t) = R_j(t) + E_j(t) + A_j(t) + I_j(t) + N_j(t) \tag{S1}$$

where R_j are recurrent internal connections within the component, E_j are episodic associations within the component, and A_j are associations from the other components of the model. I_j is external input to the node and N_j is noise. Each of these are calculated in the following way:

$$R_j(t) = \sum_i (1 - d_{ij}) w_{ij} f(x_i(t)) \tag{S2}$$

where i ranges over the nodes within the component.

$$E_j(t) = \sum_i (1 - d_{ij}) v_{ij} f(x_i(t - \tau)) \tag{S3}$$

Here τ is a time constant that sets the prediction interval for the association. Only a single delay was used here for simplicity.

$$A_j(t) = \sum_k (1 - \delta_{kj}) u_{kj} f(x_k(t)) \tag{S4}$$

where k ranges over the nodes of the other memory components. w_{ij} is the strength of the synapse between node i and j, d_{ij} is the corresponding synaptic depression. v_{ij} is the strength of the episodic associations and u_{kj} are the strengths for the external associations with δ_{ij} as the corresponding synaptic depression.

The output of each node is given by the function $f(x) = x$ that is clipped at 0 and 1. The synaptic depression changes according to,

$$\dot{d}_{ij}(t) = \alpha(1 - d_{ij}(t)) f(x_i(t)) |w_{ij}| - \beta d_{ij}(t). \tag{S5}$$

where α is the depression rate and β is the depression decay. The same dynamics is followed by δ.

Where

The output from the where-component follows a winner-take-all rule. Only one of the nodes gives an output at each time. This implements the idea that a stimulus coded in the what-system can only be at one location at a time. In all other respects, the where-system follows the same equations (1-5) as the what-component. In the current implementation, the winner is computed directly rather than relying on lateral inhibition.

Working Memory

Like the other components, the working memory is a fully connected network, but the dynamics is more elaborate. The activity of each working memory node x_i is assumed to maintain its activity once it has been activated, but decreases each time a new memory node is activated by the input. The activity levels of these nodes thus code for the recency of the corresponding input. The decay when a new node is recruited is given by,

$$\Delta x_i(t) = \lambda x_i(t), \tag{S6}$$

where $\lambda = 0.9$ is a decay constant.

The output from a working memory node is set to 1 when the node is activated by its input. Only the node with the highest activation, that is, the one with the best match between its input and stored pattern is activated in this way. When the working memory system receives a reset signal (see below), it is only the output, and not the internal state, that is reset to allow it to retain its memory state.

Learning

For the what and where components of the network, the number of co-occurring inputs n_{ij} for two nodes i and j are counted. The value of n_{ij} is increased by one every time nodes i and j simultaneously receive an input. The weights w_{ij} are subsequently set according to,

$$w_{ij} = n_{ij}/n_{ii} - n_{jj}/n \tag{S7}$$

where n is the total number of presented input patterns. This can be seen as a variant of Hebb's rule that takes into account how likely a node is to be active. The same learning rule is used for the weights u and v

except that for v, the input to the node j τ time steps ago is used instead. This particular choice of learning equation is not critical to the function of the models, and alternative learning rules could also be used.

The working memory component uses a different learning rule where a new node j is recruited for each pattern that is stored. The synaptic weights w_{ij} are directly set to the input to the working memory, that is,

$$w_{ij} = f(x_i) \tag{S8}$$

where i ranges over the nodes of the what and where networks.

Novelty, Surprise and Reset

Novelty, or surprise, is calculated as part of the attentional layer (Fig. 1) for each node with external input as the difference between the external input I_i and the internal activity pattern $f(x_i)$, that is,

$$s_i = |I_i - f(x_i)|. \tag{S9}$$

The total surprise for the complete memory systems is thus,

$$S = \sum_i s_i. \tag{S10}$$

When the surprise is above a threshold θ, a reset signal is sent to each of the memory components that sets its state to $x_i = 0$ for all i for the what and where systems, and resets the output of the working memory system.

Simulations

In the simulations, the networks were first trained on the relevant stimuli as described above in the learning section. The training phase was followed by the reported simulations. A minimal network was used in which 20 nodes were used for each of the memory systems. Each of the stimuli in the simulations were assigned a random binary patterns with 40% probability for a 1.
